# Computational Investigation of the Hemodynamic Effects of the Location of a Re-Entry Tear in Uncomplicated Type B Aortic Dissection

**DOI:** 10.3390/bioengineering11111085

**Published:** 2024-10-29

**Authors:** Eunji Kim, Sung Woon Chung, Up Huh, Seunghwan Song, Chung Won Lee, Il Jae Wang, Chanhee Song, Tae Sik Goh, Jong-Hwan Park, Dongman Ryu

**Affiliations:** 1 Department of Thoracic and Cardiovascular Surgery, Pusan National University School of Medicine and Biomedical Research Institute, Pusan National University Hospital, Busan 49241, Republic of Korea; kej2683@gmail.com (E.K.); sungwoon@pusan.ac.kr (S.W.C.); tymfoo82@gmail.com (U.H.); song77.sh@gmail.com (S.S.); vasculardoctorlee@gmail.com (C.W.L.); 2 Department of Emergency Medicine, Pusan National University School of Medicine and Biomedical Research Institute, Pusan National University Hospital, Busan 49241, Republic of Korea; jrmr9933@gmail.com; 3 Medical Research Institute, Pusan National University, Yangsan 50612, Republic of Korea; chsong0125@gmail.com; 4 Department of Orthopedic Surgery, Pusan National University School of Medicine and Biomedical Research Institute, Pusan National University Hospital, Busan 49241, Republic of Korea; taesikgoh@gmail.com; 5 Department of Convergence Medicine, Pusan National University School of Medicine, Yangsan 50612, Republic of Korea; parkj@pusan.ac.kr; 6 Convergence Medical Institute of Technology, Pusan National University Hospital, Busan 49241, Republic of Korea

**Keywords:** aortic dissection, uncomplicated type B aortic dissection, re-entry tear location, fenestration, computational fluid dynamic

## Abstract

This study aimed to examine the hemodynamic modifications in uncomplicated type B aortic dissection in relation to the location of re-entry tears using a computational fluid dynamics simulation. The geometry of uncomplicated type B aortic dissection was reconstructed using computed tomography images. Subsequently, 10 virtual models were artificially generated with re-entry tears at various locations. The simulation results indicated that most models with re-entry tears had lower pressure and wall shear stress than those without re-entry tears. The overall pressure distribution of the true lumen was greater than that of the models without re-entry tears when the re-entry tear was placed at the end of the false lumen. Furthermore, the recirculation phenomenon in the false lumen was reduced as the re-entry tear was relocated to the distal region of the aorta. To determine whether and how to perform fenestration surgery in patients with uncomplicated type B aortic dissection, these computational results can be used as supplemental indicators. However, further validation in a larger number of patients through additional investigation is necessary.

## 1. Introduction

Aortal dissection is a common cardiovascular disease characterized by a high mortality rate and a global incidence of approximately 0.5–2.95 cases per 100,000 people per year [1,2,3]. It occurs when the aortic wall separates due to the effect of high blood pressure entering the medial wall through a rupture in the medial intake [1,2,4]. It is the result of a tear in the inner layer of the aorta, which causes blood to enter the middle layer of the aorta, creating both true and false lumens [5,6,7].

Aortic dissection has the potential to propagate in either the distal or proximal direction from the point of rupture [8]. When considering percutaneous treatment with endovascular stent grafts or surgical treatment for aortic dissection, it is important to differentiate between true and false lumens [9,10]. Before therapy, it is essential to detect the lumens that originate from significant branch vessels, such as the coronary, carotid, renal, and mesenteric arteries. When a false lumen is naturally or surgically blocked, there is a potential risk of infarction to other organs that are supplied via the false lumen [11].

The classification of aortic dissection is based on the length of false lumen propagation and the location of the intimal tear. An intimal tear primarily occurs at the location with the highest level of hemodynamic stress. It is most frequently observed in the lateral wall of the ascending aorta and in the portion immediately distal to the ligamentum arteriosus of the descending thoracic aorta. Two extensively used classification methods for aortic dissection have been established: the DeBakey and Stanford system [1,8,12,13,14]. The DeBakey system classifies aortic dissection into three types: type 1, which occurs when aortic dissection starts in the ascending aorta and extends to the aortic arch, and often further down; type 2, which occurs when aortic dissection only starts in the ascending aorta; and type 3, which occurs when aortic dissection starts in the descending aorta and extends below the aortic arch [15,16]. The Stanford system is widely used by surgeons as a straightforward criterion for determining the level of urgency, while the DeBakey system is a useful instrument for comprehending the clinical characteristics of aortic dissection [13].

Surgery is the preferred treatment for Type A aortic dissection in the management of the disease. Meanwhile, Type B aortic dissection (TBAD) is classified into two subtypes: complicated and uncomplicated. Complicated TBAD is treated using thoracic endovascular aortic repair (TEVAR) and has a high mortality rate after surgery. In contrast, for uncomplicated TBAD, drug therapy should be prioritized to maintain optimal heart rates and blood pressure control [17,18,19]. Recent medical data indicate that a considerable proportion of patients with uncomplicated acute TBAD treated with medication die because of aneurysm growth and subsequent aortic complications such as organ or peripheral ischemia. This highlights the necessity of surgical intervention in such cases [17,20,21,22,23]. Surgery is necessary for patients with stable chronic aortic dissection if they experience symptoms or if the diameter of the aorta measures 6 cm or greater. Critical postoperative complications may arise or persist and can occur even after medical intervention [24]. Aortic fenestration techniques can be used to relieve or stabilize certain ischemic complications in the viscera or lower extremity [25,26]. In cases of aortic dissection, when there are no previous fenestrations in essential locations, it is necessary to generate an opening in the septum between the true and false lumens. These openings can be utilized during the acute phase to address malperfusion and for the insertion of a thoracic endovascular aortic repair (TEVAR) endograft or frozen elephant trunk (FET) into the false lumen. These can also be used during the chronic phase when target vessels arise from the false lumen in fenestrated-branched endovascular aneurysm repair (FB-EVAR) [27,28,29].

Aortic dissection is classified based on the patient’s medical condition and other environmental variables, and the treatment strategy can be customized accordingly. However, complications still occur following the treatment of patients with aortic dissection. To address these problems, it has been proposed to obtain data about the vascular geometry, mechanical characteristics of the aortic wall, and effects of hemodynamics [5,30]. Medical image analysis methods such as computed tomography (CT) may accurately represent the anatomical composition associated with the true and false lumens of the aortic dissection. However, these techniques are insufficient to provide information regarding hemodynamic effects [31]. Hence, numerous researchers have consistently investigated vascular diseases using computational fluid dynamics (CFD) to examine the variations in blood flow patterns within blood vessels. CFD can help comprehend the hemodynamic effects of aortic dissection, thereby facilitating effective patient management and preventing potential complications [31].

Tse et al. (2011) conducted CFD investigations that demonstrated that vortices are formed in the thoracic aorta because of variations in the cross-sectional area and curvature of the vessel [6]. CFD modeling has been confirmed to be an effective approach for obtaining information on blood flow patterns, wall pressures, and wall shear stresses, which is helpful for management planning and gaining insight into the growth of dissecting aneurysms. Cheng et al. (2013) demonstrated that the volume of circulating flow in the false lumen is influenced by the location and dimensions of the proximal rupture in the aorta [32]. In addition, it has been proposed that implementing computer simulations to study aortic hemodynamics could provide valuable predictive information for identifying complicated acute TBADs. Zhu et al. (2023) correlated CFD analysis results to 4D MRI data and assessed the vascular hemodynamics of aortic dissection with both techniques [33].

Multiple experimental and numerical studies have examined the effects of tear dimensions, location, and vascular patency within the false lumen on blood flow characteristics based on hemodynamics. Tsai et al. (2008) demonstrated that diastolic false lumen pressure from in vitro tests was significantly increased by reducing the dimensions of the proximal tear and removing the distal tear [34]. This is an essential factor in the regulation of both the inflow and outflow of blood. Similarly, Cheng et al. (2010) indicated that the volume of blood flowing into the false lumen is dependent on the location and dimensions of the proximal and distal tears, which in turn influence the expansion and rupture of the false lumen [35]. Tang et al. (2012) and Karmonik et al. (2011) conducted studies that examined variations in tear numbers with and without re-entry tears [36,37].

Despite various studies on the topic, the hemodynamic differences associated with fenestration surgery, namely, the creation of an exit tear, have not been explored in relation to the specific location of the surgical site. Examination of the surgical location in fenestration surgery can assist in anticipating the pressure difference between the true and false lumens post-surgery, prognosticate the result in the absence of treatment, and facilitate treatment planning. Hence, this study aimed to examine the influence of the location of an exit tear in the aorta on the distribution of blood flow, pressure, and wall shear stress (WSS) in patients with aortic dissection.

## 2. Methods

### 2.1. Geometry Reconstruction

CT images of patients diagnosed with uncomplicated TBAD were used to extract anatomical information. This study was conducted in accordance with the Declaration of Helsinki and approved by the Institutional Review Board of Pusan National University Hospital (IRB No. 2309-018-130).

**Figure 1:**
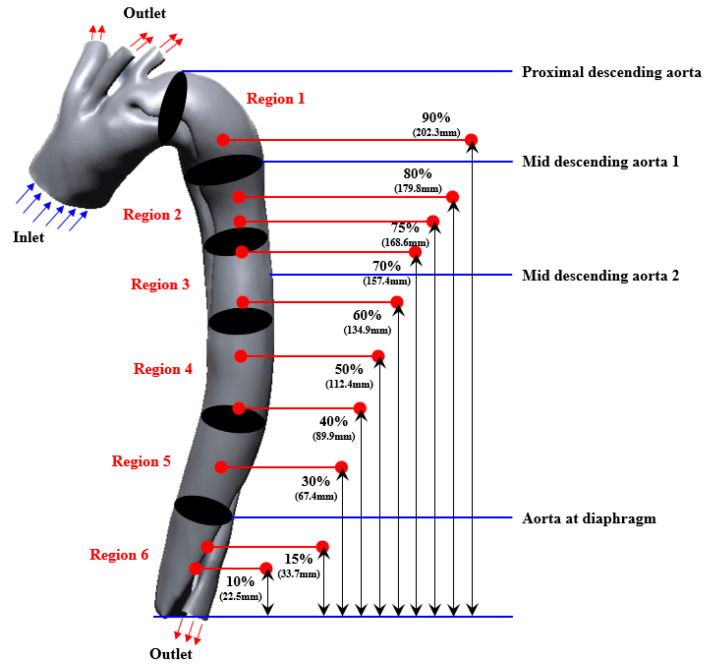
Reconstructed geometric model of aortic dissection and the different scenarios for simulation.

The model geometry was constructed using the initial CT images, which were approximately 1159 slices and 2 mm thick, with a spacing of 1 mm and a pixel resolution of 0.86 mm. The images represent the entire aorta, as illustrated in Figure 1. The geometry of the dissected aorta was reconstructed from the contours of the segmented lumen regions in consecutive CT slices after these CT images were imported into the open-source medical imaging software 3D Slicer v5.2.2. In addition, the model surface was smoothed before mesh generation.

The blue and red arrows indicate the inlet and outlet of the blood flow in the model geometry, respectively (Figure 1). The false lumen progresses along the lateral aspect of the thoracic descending aorta and subsequently spirals downward around the true lumen of the mid-descending aorta. It subsequently twisted toward the right posterior aspect of the aorta and lateralized toward the distal aspect of the abdominal aorta.

This study presents the reconstruction of geometric models that include re-entry tears at various locations below the existing entry tear to determine the most suitable location for fenestration. The anatomical landmarks illustrated in Figure 1 were used to establish the re-entry interval [38]. A total of 11 cases (Figure 1) were examined: (i) an entry-only tear (case 1); (ii–xi) a re-entry tear with a diameter of 6.4 mm that is situated 22.5 (case 2)/33.7 (case 3)/67.4 (case 4)/89.9 (case 5)/112.4 (case 6)/134.9 (case 7)/157.4 (case 8)/168.6 (case 9)/179.8 (case 10)/202.3 (case 11) mm proximal to the floor of the false lumen. According to Keramati et al. (2020), blood flow is more stable when the re-entry tear diameter is 10 mm rather than 5 mm [39]. Nevertheless, a re-entry tear diameter of 10 mm was difficult to establish in this study after confirming the long and short axes of the false lumen. Consequently, the re-entry tear diameter was determined to be 6.4 mm, which was 50% of the short axis of the false lumen.

### 2.2. Flow Simulation and Boundary Conditions

Simulations of the series were conducted using the ANSYS FLUENT package 2024 R2 (ANSYS, Inc., Canonsburg, Pa, USA). The blood flow in the model was considered laminar because the maximum Reynolds number (Re_max_) was below the critical Reynolds number (Re_c_) range [40,41]. The blood is assumed to be Newtonian, incompressible, and homogeneous, with a dynamic viscosity of 3.71 mPa.s and a density of 1060 kg/m^3^ [6]. The aortic wall was assumed to be a rigid body with no slip.

**Figure 2:**
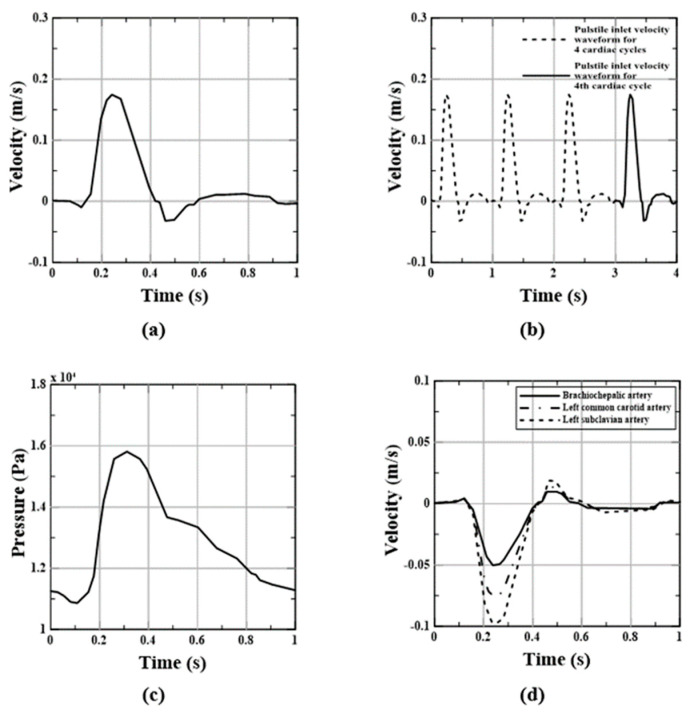
Pulsatile (**a**) inlet velocity waveform, (**b**) cardiac cycles for inlet velocity waveform, (**c**) outlet pressure waveform, and (**d**) outlet velocity waveform at the three aortic branches [6,42,43].

The flat velocity profiles applied to the ascending aortic inlet with a pulsatile flow were derived from Tse et al. and Olufen et al. [6,42], as shown in Figure 2a. The flow outlets were set up at the left subclavian artery, left common carotid artery, brachiocephalic artery, and descending aorta, all of which had different diameters. It was assumed that 5% of the flow was diverted to each branch outlet, as observed in a healthy aorta (Figure 2d), and pulsatile inlet velocity profiles were applied to each of the three aortic arch branches by adjusting the outlet. The outlet conditions are indicated by negative velocities. In contrast, the descending aortic outlet was subjected to pulsatile pressure waveforms, as shown in Figure 2c [6,42].

### 2.3. Governing Equations

In this study, numerical simulation of pulsatile blood flow through the aorta model was conducted by solving mass continuity and Navier–Stokes equation below.

(1) ∇·v=0

(2) $$\frac{\partial }{\partial t}\left(\rho v\right)+\rho \left(v\cdot \nabla \right)v=-\nabla p+\nabla \cdot \tau$$

where the symbol ρ, **v,** and *p* represent the blood density, velocity vector, and pressure at time *t*, and $\tau =\mu (\nabla v+{\left(\nabla v\right)}^{T})$ is the viscous stress tensor of Newtonian fluid, where μ is the dynamic viscosity. Equations (1) and (2) were discretized with the second-order upwind scheme. The finite volume method in the ANSYS FLUENT package is used to run the simulations.

### 2.4. Computational Method

The computational efficacy is enhanced in this study through the application of the Pressure Implicit with Splitting of Operators (PISO) pressure–velocity term coupling method, which is characterized by a higher degree of approximation between the pressure and velocity corrections. Furthermore, the periodic flow characteristics were simulated based on four cardiac cycles, and the results presented in this study were based on the fourth cardiac cycle. To demonstrate the systolic and diastolic phases, the simulation results were derived at two points: t = 3.25 s (peak systole) and t = 3.47 s (early diastole) during the examination stage. Furthermore, a comparative analysis was conducted between the series of simulation results categorized into six regions, as shown in Figure 1.

### 2.5. Mesh Sensitivity Test

Quadratic tetrahedron-type elements were constructed using the meshing tool of the ANSYS FLUENT package, with a total of 403,234 elements. Step-by-step simulations were conducted based on the number of elements, and a mesh sensitivity test was performed for Case 1. Table 1 lists the wall shear stress values at t = 3.25 s and t = 3.47 s, respectively. The number of elements in this study was set to 400,000 or more because the difference in the WSS value according to the number of elements was less than 5.0% when the number of elements exceeded 400,000.

## 3. Results

### 3.1. Flow Distribution

**Figure 3:**
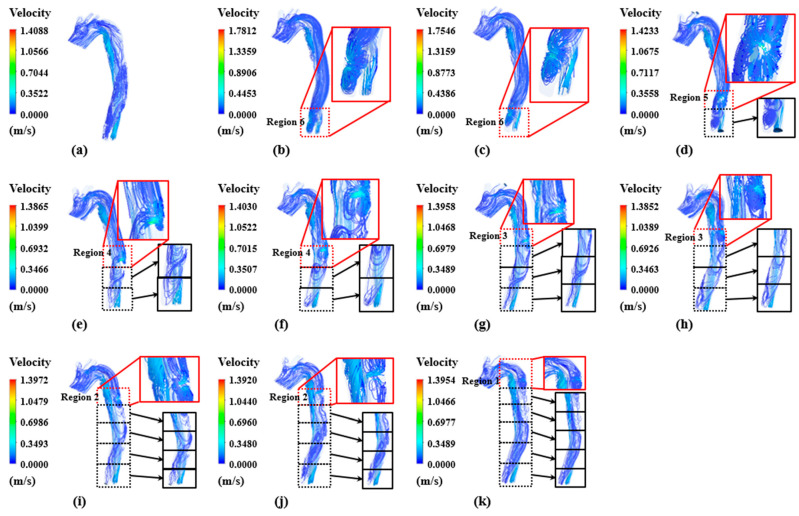
Flow distribution in the proximal and distal locations of true and false lumens of (**a**–**k**) cases 1–11 during the systolic phase.

**Figure 4:**
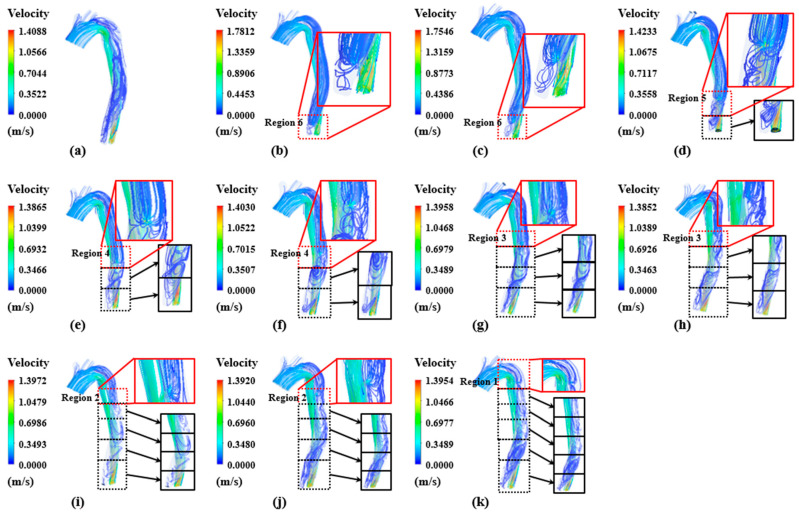
Flow distribution in the proximal and distal locations of true and false lumens of (**a**–**k**) cases 1–11 during the diastolic phase.

The overall velocity streamline-based flow distribution, as illustrated in Figure 3 and Figure 4, includes regions near the entry and re-entry tears between the true and false lumen during systole and diastole, respectively. Low flow velocities were consistently observed in the proximal false lumen at all time points despite flow pulsatility. In some cases, slow flow recirculation was observed in the region close to the entry tear. A re-entry tear enables the return of a portion of blood that passes through the entry tear to the true lumen. Flow recirculation was observed in the area below the re-entry tear because of the remaining blood that did not return to the true lumen.

The blood flow velocities in the true lumen were higher than those in the false lumen during the systolic phase. In case 1, without a re-entry tear, the highest blood flow velocity of 1.66 m/s was observed in region 6 of the true lumen. However, the re-entry tear was the location with the greatest blood flow velocity in cases 2–4, whereas region 5–6 of the true lumen was the location with the highest blood flow velocity in cases 5–11.

The diastole phase was characterized by recirculation and retrograde flow in all cases, with recirculation occurring in the vicinity of the tear. In particular, blood flows into the false lumen from the true lumen, with the highest velocities observed in the vicinity of the re-entry tear, accompanied by a distinct flow jet. Subsequently, the blood returns to the true lumen via an entry tear in the proximal region.

### 3.2. Pressure Distribution

**Figure 5:**
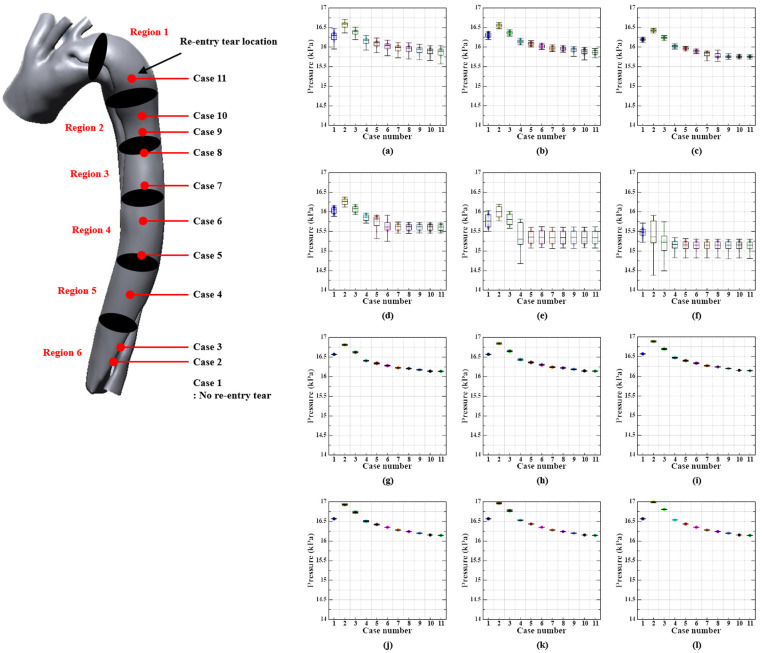
Pressure values according to the re-entry tear location in the true and false lumens during the systolic phase. First and second rows show pressure values according to the region in the true lumen ((**a**) T-Region 1, (**b**) T-Region 2, (**c**) T-Region 3, (**d**) T-Region 4, (**e**) T-Region 5, (**f**) T-Region 6), while the third and fourth rows show pressure values according to the region in the false lumen ((**g**) F-Region 1, (**h**) F-Region 2, (**i**) F-Region 3, (**j**) F-Region 4, (**k**) F-Region 5, (**l**) F-Region 6).

**Figure 6:**
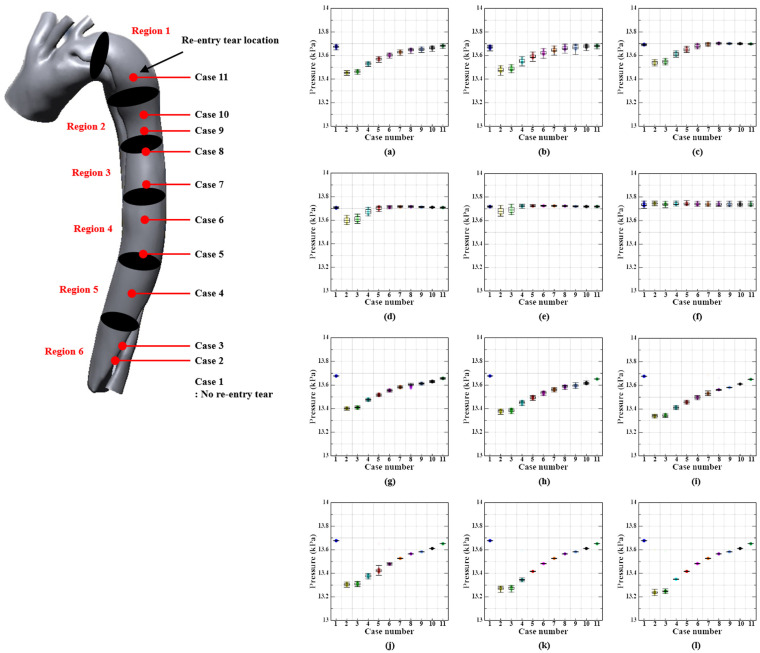
Pressure values according to the re-entry tear location in the true and false lumens during the diastolic phase. First and second rows show pressure values according to the region in the true lumen ((**a**) T-Region 1, (**b**) T-Region 2, (**c**) T-Region 3, (**d**) T-Region 4, (**e**) T-Region 5, (**f**) T-Region 6), while the third and fourth rows show pressure values according to the region in the false lumen ((**g**) F-Region 1, (**h**) F-Region 2, (**i**) F-Region 3, (**j**) F-Region 4, (**k**) F-Region 5, (**l**) F-Region 6).

Figure 5 and Figure 6 illustrate the pressure values for the true and false lumens in cases 1–11 during the systolic and diastolic phases, respectively, as determined by the location of the re-entry tear. The pressure values for the re-entry tear locations were not distinguishable; however, they presented a gradual decrease in the direction of flow, which is consistent with the findings of previous studies [6,43]. Region 1 experiences the maximum pressures of 16.3, 16.58, 16.4, 16.17, 16.11, 16.04, 15.99, 15.97, 15.94, 15.92, and 15.90 kPa in the true lumens of cases 1–11, respectively, during the systolic phase, while region 6 experiences the lowest pressures. In cases 1–11, the pressure differences between the true lumen inlet and outlet were 0.82, 1.22, 1.18, 1.00, 0.96, 0.89, 0.84, 0.82, 0.79, 0.77, and 0.75 kPa, respectively.

True lumen pressures were consistently slightly lower than those of the false lumen in region 1, the proximal portion of the aortic dissection, during the systole phase, with a difference of approximately 0.22–0.27 kPa. The difference increases to approximately 0.99–1.64 kPa as it proceeds to region 6, the distal part of the aortic dissection. The pressure values for the true and false lumens in regions 1–6 were slightly higher in Case 2 than in the other cases. The slope of the pressure difference was confirmed to be a gentle curve as the re-entry tear proceeded toward the proximal part of the aortic dissection in comparative investigations of the pressure differences between the cases.

In contrast with the results in the systolic phase, the pressure values of the true lumen in all regions were slightly higher than those of the false lumen in the diastolic phase. In particular, the pressure differentials between the true and false lumens in the distal aorta were the highest in cases 2–5, with a range of 0.32–0.51 kPa. The pressure values for the true and false lumens in all regions were the lowest in Case 2, in contrast to the results in the systolic phase. In addition, comparative studies of the pressure values between cases have confirmed that the slope of the pressure difference is the opposite of that in the systolic phase, as the re-entry tear progresses toward the proximal aortic dissection.

### 3.3. Wall Shear Stress Distribution

The aortic outlet region exhibited the highest WSS during the systolic phase, except in cases 2 and 4. In cases 2–4, the re-entry tears had the highest WSS. In all cases, the re-entry tear was the location with the highest WSS during the diastolic phase. Nevertheless, WSS values in the systolic phase were higher than those in the diastolic phase.

**Figure 7:**
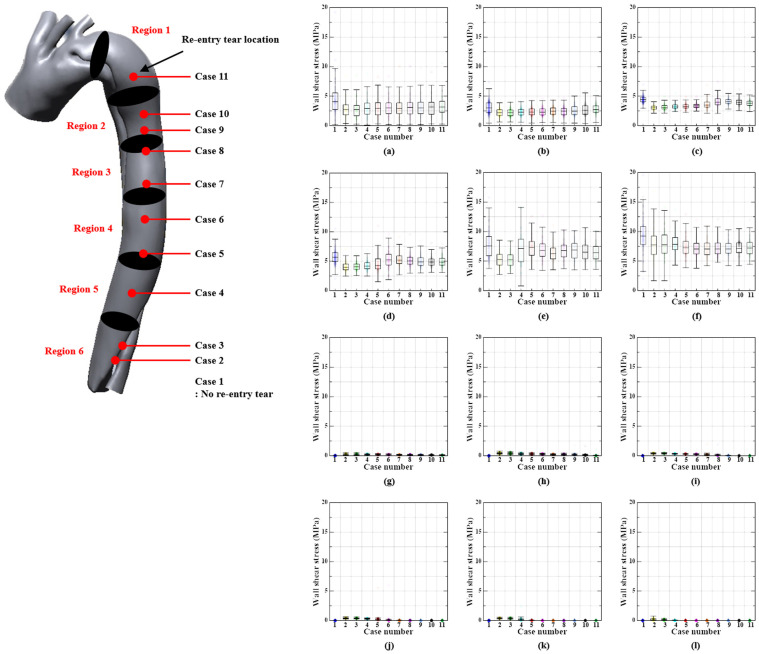
WSS according to re-entry tear location in the true and false lumens during the systolic phase. First and second rows show the WSS values according to region in true lumen ((**a**) T-Region 1, (**b**) T-Region 2, (**c**) T-Region 3, (**d**) T-Region 4, (**e**) T-Region 5, (**f**) T-Region 6), while the third and fourth rows show the WSS values according to region in false lumen ((**g**) F-Region 1, (**h**) F-Region 2, (**i**) F-Region 3, (**j**) F-Region 4, (**k**) F-Region 5, (**l**) F-Region 6).

**Figure 8:**
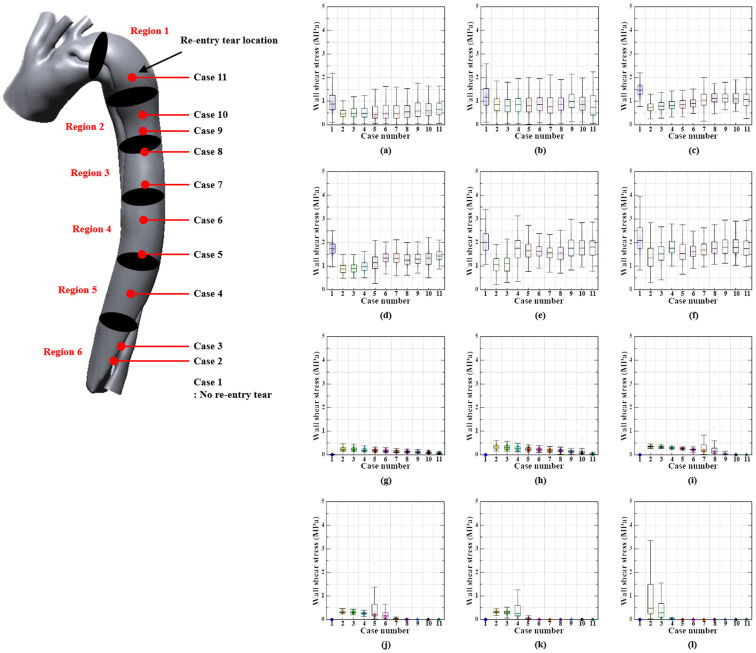
WSS according to re-entry tear location in the true and false lumens during the diastolic phase. First and second rows show the WSS values according to region in true lumen ((**a**) T-Region 1, (**b**) T-Region 2, (**c**) T-Region 3, (**d**) T-Region 4, (**e**) T-Region 5, (**f**) T-Region 6), while the third and fourth rows show the WSS values according to region in false lumen ((**g**) F-Region 1, (**h**) F-Region 2, (**i**) F-Region 3, (**j**) F-Region 4, (**k**) F-Region 5, (**l**) F-Region 6).

Figure 7 and Figure 8 show the median WSS values of the true and false lumen in relation to the re-entry location during the systolic and diastolic phases. In the systolic phase, the aortic outflow region had the median value of the highest WSS. In contrast, in the diastolic phase, the median values for the highest WSS were found in region 2. The WSS values in cases with re-entry tears were slightly lower than those in cases without re-entry tears, although the WSS values in all cases did not differ significantly.

## 4. Discussion

### 4.1. Flow Distribution

In all cases, the entry tear divided blood into the true and false lumens during the systolic phase. Blood flowed at a high velocity in the true lumen near the entry tear, according to the results of the series of CFD simulations. This is in accordance with the findings of Naim et al. (2014), Dillon-Murphy et al. (2016), and Zhu et al. (2021) [43,44,45]. In cases 2–11, the re-entry tear enabled the return of blood from the false lumen to the true lumen. As reported by Dillon-Murphy et al. (2016) and Zhu et al. (2021) [44,45], the vortex phenomenon was observed in Case 1, in which blood entered the false lumen through blood recirculation. Furthermore, in cases 2–11, the blood that could not exit through the re-entry tear was recirculated under the re-entry tear.

The blood flow velocity decreased during the diastolic phase compared with that during the systolic phase, which led to a general variation in the pressure distribution. During this phase, blood flows from the true lumen to the false lumen through the re-entry tear, reversing the direction of flow from the systolic phase. The false lumen below the re-entry area revealed blood recirculation. These flow phenomena can potentially induce recirculation and blood–wall interactions. Platelet precipitation has been reported to be induced by recirculation in particular [46,47], which may result in thrombus formation.

The re-entry tear location in the present study did not significantly influence the direction of the overall blood flow or flow velocity, except for cases 1–3. Nevertheless, it offers information on the potential locations of recirculation that could occur as a result of the re-entry tear location during diastole and systole. This study demonstrated that the closeness of the re-entry tear to the proximal aorta results in increased recirculation in the area below the re-entry tear, which can have a negative impact on systole and diastole. However, it is important to reduce the pressure and WSS values resulting from re-entry tears in comparison with cases in which there are no re-entry tears. To determine the optimal re-entry tear environment, we will implement a series of simulations that incorporate variables such as the location, angle, dimensions, and number of re-entry tears.

### 4.2. Pressure Distribution

The investigation conducted by Rudenick et al. (2010) verified that lumen expansion and rupture are correlated with an increase in pressure within the false lumen [5]. Moreover, the pressure in the true lumen decreases in accordance with the location along the direction of blood flow, whereas the pressure in the false lumen varies only minimally [43].

The results of the series simulation in this study were comparable to those reported by Tse et al. (2011) and Naim et al. (2014) [6,43]. They reported that the true lumen pressure was higher than the false lumen pressure in the proximal aortic region during the systolic phase, whereas the distal aortic region experienced the opposite phenomenon. Nevertheless, the pressure in the false lumen was slightly higher than that in the true lumen in this investigation. This is believed to be a consequence of divergent results caused by the dimension or angle of the blood entry tear into the false lumen in the proximal aortic region [45]. In addition, the pressure difference between the true lumen and false lumen is at its highest in the area below the re-entry tear in all cases except cases 2 and 3, which may result in the expansion and rupture of the false lumen [6]. In cases 2 and 3, the true lumen pressure was lower than that of the false lumen because the re-entry tear was located where the maximum blood flow velocity occurred.

Furthermore, the true lumen pressure was slightly higher than the false lumen pressure in all patients during the diastolic phase. These findings could be useful as fundamental data for further investigations during fenestration surgery, which may involve the consideration of additional re-entry tears.

### 4.3. WSS Distribution

In cases 2–4, the location of maximum WSS occurrence was associated with re-entry tears, whereas in the other cases, it was located in the aortic outflow region, which was associated with the location of the highest blood flow velocity. Furthermore, the WSS in this study was greater than 1–7 Pa in the arterial wall of a healthy person, which could encourage the rupture of the arterial wall and expansion of re-entry tears [47]. However, the WSS values of patients with re-entry tears were lower than those of patients without re-entry tears. This suggests that the WSS can be reduced by re-entry tears.

Naim et al. (2014) found that the proximal part of the aorta demonstrated low WSS owing to several additional re-entry tears, whereas the distal part presented moderate and concentrated time-averaged WSS [43]. It is hypothesized that the WSS value can be modified by varying the location of additional re-entry tears, as indicated in this study.

### 4.4. Clinical Value

It is difficult to determine the most effective treatment method for patients with uncomplicated TBAD due to the ongoing debate concerning its treatment. One of the treatments for patients with uncomplicated TBAD is the implantation of an endovascular stent, which covers the entry tear to prevent blood from entering the false lumen. This treatment induces thrombosis to close the false lumen; however, it has been reported to have a higher mortality rate than that in patients with an open false lumen [37]. Furthermore, a study conducted by Tsai et al. (2008) confirmed that the pressure within the false lumen was substantially increased by the obstruction of entry or re-entry tears [34].

In contrast, Naim et al. (2014) reported that the pressure in the false lumen decreased in relation to the number of re-entry tears, thereby emphasizing the importance of fenestration surgery as a treatment for uncomplicated TBAD [43]. Fenestration treatment can prevent thrombosis and relieve malperfusion syndrome by encouraging the return of blood from the false lumen to the true lumen through the generation of re-entry tears in the false lumen [48,49]. Nevertheless, this treatment lacks clinical evidence and carries the risk of false lumen expansion and rupture, in addition to an increase in blood flow to the false lumen. Therefore, further research is required [49]. This study provides fundamental data for identifying blood flow in the re-entry tear location and offers a supplementary index to determine the optimal location when two to three re-entry tears are considered, thereby addressing the weaknesses of fenestration treatment.

### 4.5. Limitations

The aortic wall was assumed to be a rigid body in the present work because the vessel’s flexibility is significantly decreased by the lack of elastin, as reported in a study conducted by Boussel et al. (2009) [50]. In addition, the flow was assumed to be laminar. This study was conducted following the methodology described by Tse et al. (2011), Lam et al. (2008), Morris et al. (2005), and Naim et al. (2014) [6,40,41,43]. Turbulent flow studies will be performed in the future to verify the reliability of re-entry tear studies.

Simulations were conducted on one specimen in this investigation without considering the various geometric configurations of the entry tear. It is difficult to consider various specimens because the incidence of similar types of diseases is low and data obtained from low-resolution images are excluded [51]. Therefore, the geometric models, number, and location of additional re-entry tears should be considered to determine the optimal conditions for fenestration surgery to generate re-entry tears. This study provides an initial phase in the investigation of the optimal fenestration surgical techniques. Our goal is to determine the optimal fenestration surgical technique using additional studies. In the future, more reliable studies will be conducted by obtaining a variety of parameters such as physiological variables, medical factors, and numerical model characteristics.

## 5. Conclusions

The results of the analysis of blood flow, pressure, and velocity distribution in the systolic and diastolic phases in relation to the location of re-entry tears are presented in this study. Recirculation phenomena have been confirmed to occur under re-entry tears. However, the pressure and WSS distributions in cases of re-entry tears are lower than those in cases without re-entry tears. In cases 2 and 3, the recirculation area under the re-entry tear had small dimensions, which could be considered for deciding about fenestration. Furthermore, the fenestration surgery technique would be considerably improved by the inclusion of re-entry tears in cases 4–11, which have a low pressure and WSS distribution. These findings provide fundamental data for the development of ancillary treatment strategies for uncomplicated TBAD.

## Figures and Tables

**Table 1:** Results of the mesh sensitivity test for maximum wall shear stress occurrence.

Number of Elements	Time 3.25 s (Peak Systole)	Time 3.47 s (Early Diastole)
	Maximum WSS (Pa)	Maximum WSS (Pa)
8114	8.69	0.04
110,280	31.38	7.75
201,230	17.95	1.32
303,571	22.33	1.93
403,234	26.99	1.95
450,667	24.03	1.76
501,162	26.44	1.76

## Data Availability

The original contributions presented in the study are included in the article, further inquiries can be directed to the corresponding author.
